# Advances in robotic lung transplantation: development and validation of a new surgical technique in animal models

**DOI:** 10.1093/icvts/ivad179

**Published:** 2023-11-06

**Authors:** Fernando Ascanio, Iñigo Royo-Crespo, Joel Rosado, Leire Sánchez, Laura Romero, David Durán-Rey, Francisco Sánchez-Margallo, Alberto Jauregui

**Affiliations:** Department of Thoracic Surgery and Lung Transplantation, Hospital Universitario Vall d’Hebron, Barcelona, Spain; Department of Thoracic surgery, Hospital Universitario Miguel Servet and Hospital Clínico Lozano Blesa, Zaragoza, Spain; Department of Thoracic Surgery and Lung Transplantation, Hospital Universitario Vall d’Hebron, Barcelona, Spain; Department of Thoracic Surgery and Lung Transplantation, Hospital Universitario Vall d’Hebron, Barcelona, Spain; Department of Thoracic Surgery and Lung Transplantation, Hospital Universitario Vall d’Hebron, Barcelona, Spain; Laparoscopy Unit, Jesús Usón Minimally Invasive Surgery Centre, Cáceres, Spain; Laparoscopy Unit, Jesús Usón Minimally Invasive Surgery Centre, Cáceres, Spain; Department of Thoracic Surgery and Lung Transplantation, Hospital Universitario Vall d’Hebron, Barcelona, Spain

**Keywords:** Lung transplantation, Robotic surgery, Minimally invasive

## Abstract

The objective of this study was to describe a novel minimally invasive robotic video-assisted approach for lung transplantation, utilizing a minimally invasive technique with a subxiphoid incision, in an animal experimentation model. Two left robotic-assisted single lung transplants were performed in sheep using a robotic surgical system. A subxiphoid incision was made, and robotic ports were inserted into the thoracic cavity for dissection and anastomoses of the bronchus, artery, and pulmonary veins. The integrity of anastomoses was evaluated, and procedural details were recorded. Both animals survived the procedure, with a mean duration of 255 min and a mean console time of 201 min. Anastomoses were performed without complications, and the closed-chest approach with a subxiphoid incision proved successful in preventing gas leakage. The novel approach demonstrated improved exposure and workflow compared to existing techniques. The minimally invasive robotic video-assisted approach for lung transplantation utilizing a closed-chest technique with a subxiphoid incision appears safe and feasible in an animal experimentation model. Further studies in the clinical setting are warranted to establish its feasibility and safety in human lung transplantation. This approach has the potential to offer benefits over the traditional Clamshell incision in lung transplantation procedures.

## INTRODUCTION

Lung transplantation is considered the gold standard treatment for patients with end-stage lung disease. Despite the advancements in surgical techniques since the first successful clinical lung transplantation performed in 1963 by Dr. James D. Hardy at the University of Mississippi Medical Center and then followed by the Toronto Lung Transplant Group [1], the use of a bilateral anterolateral thoracotomy with sternal section (clamshell incision) or anterior thoracotomies remains widely used approach for bilateral lung transplantation.

However, this highly invasive approach is associated with several detrimental outcomes [2], including increased systemic inflammation, postoperative pain, phrenic nerve paresis leading to respiratory dysfunction, impaired wound healing, dehiscence and infection, including osteomyelitis, which can negatively impact postoperative outcomes and hinder the recovery process, thus compromising the quality of life for the patient.

Despite the widespread use of minimally invasive surgical techniques in the treatment of lung cancer, there is a lack of published literature detailing their application in lung transplantation procedures [3].

With the evolution of robotic surgery and the advances made in the surgical technique of lung transplantation, we have developed a novel minimally invasive robotic video-assisted approach for lung transplantation that may avoid the use of thoracotomy.

## TECHNIQUE

### Ethics statement

The experimental protocol was approved by the Jesús Usón Minimally Invasive Surgery Centre’s Ethical Committee for Animal Research (Reference: 015/22) and complied fully with Directive 2010/63/EU of the European Parliament on the protection of animals used for scientific purposes.

We describe the surgical technique used to perform 2 left robotic-assisted single lung transplants in an animal experimentation model (sheep).

Two single left lung retrievals were performed using a standard surgical technique, then another 2 sheep (weighing 49 and 52 kg, respectively) were anesthetized and selective right lung ventilation was achieved. The animals were positioned in a decubitus position, slightly lateralized to the right.

**Figure 1:**
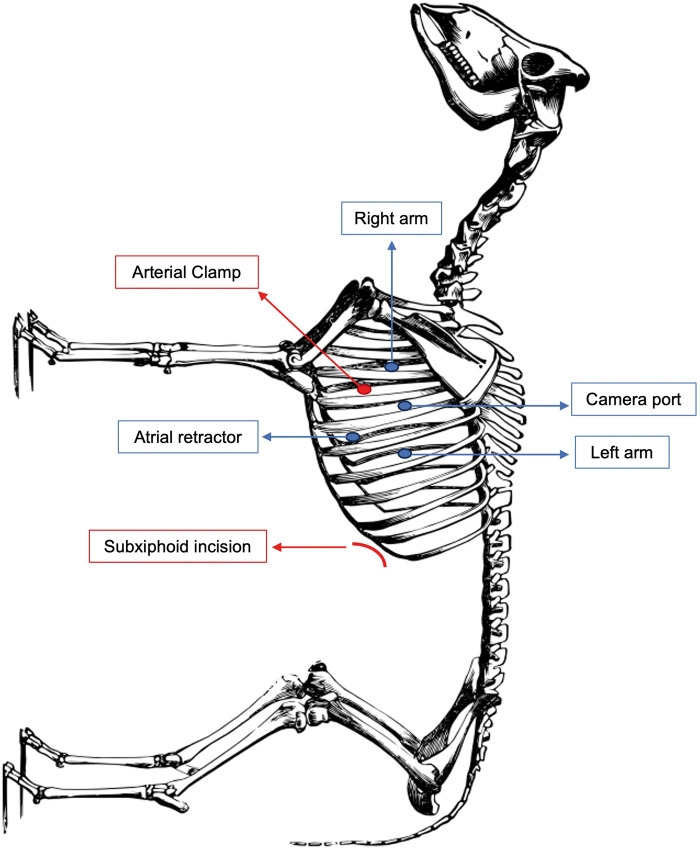
Port placement for robotic lung transplantation in sheep.

A subxiphoid 8-cm incision was made, and a flexible wound retractor (GelPort^r^) was placed. A camera port was subsequently inserted, and carbon dioxide was insufflated to the left hemithorax to maintain a pressure of 8 mmHg. A 30° endoscopic camera of the Da Vinci robotic surgical system was inserted into the left thoracic cavity, and an 8-mm port was placed in the 5th intercostal space with anterior axillary line to be used as the camera port. Subsequently, 2 instrument ports (12 mm) were inserted into the 3rd and 7th intercostal spaces in the anterior axillary line and another 8-mm robotic port was placed more proximally to the sternum in the 6th intercostal space to facilitate exposure by mediastinal retraction. The subxiphoid incision was also utilized as an auxiliary port to assist the surgeon in the removal of the native lung after pneumonectomy and introduction of the graft (Fig. 1).

The robotic surgical system was brought to the operating table from the right side, and the right and left robotic arms were inserted into the left pleural cavity. The pulmonary hilum was dissected, the pulmonary artery and veins were isolated and transected with robotic staplers entered from the left arm and the left main bronchus was transected with monopolar robotic scissors. The lung was removed through the subxiphoid port and then the hilum was prepared for the implantation. The lung graft was inserted into the cavity through the subxiphoid incision, taking advantage of the high distensibility of the soft tissues and ensuring proper lung deflation, thus avoiding any compression issues for the graft.

The anastomoses of the bronchus, artery and pulmonary veins were performed according to generally accepted techniques, including a running suture for the bronchial anastomosis with a barbed 4–0 monofilament absorbable running suture. Once the bronchus anastomosis was completed, through the mediastinal retractor port, another Satinsky clamp was placed in the left atrium and the anastomosis of the atrial labrum was performed to the superior pulmonary vein (for anatomical reasons of the sheep) by continuous suture with 3–0 polypropylene, without everting the atrial cuff due to our primary focus was to demonstrate the feasibility and reproducibility of the surgical technique. In doing so, we may have overlooked certain aspects, such as the importance of everting the atrial cuff, which is essential in our routine clinical practice. A Satinsky clamp was then placed in the pulmonary artery through an auxiliary incision in the 4th intercostal space, midclavicular line. The artery anastomoses were made using 5–0 expanded polytetrafluoroethylene (ePTFE) running suture. Through the subxiphoid incision, another Satinsky clamp was placed in the left atrium and the anastomosis of the atrial labrum was performed by continuous suture with 3–0 polypropylene. During the clamp placement, we noticed a small gas leak; however, we found that by placing gauze around the ports, we were able to maintain intrathoracic pressure and minimize losses without significantly enlarging the wounds. After anastomoses were completed, clamps were gradually removed, anastomoses were checked for bleeding and air leaks and the lung was reinflated and re-expanded successfully (Video 1). In the video, it is observed small tears that occurred during the anastomoses and that later during declamping did not cause any significant problems. We believe that the great visualization of the robotic system with magnification and 3D allows us to see things that would be imperceptible to the naked eye. As part of our standard practice in lung transplants, we performed a progressive declamping technique and applied pressure using gauze initially, followed by the use of a human thrombin-based haemostat. These measures were effective in preventing any significant bleeding from the anastomosis site. During the surgery, we did not require any additional haemodynamic support such as Extracorporeal membrane oxygenation (ECMO) or cardiopulmonary bypass.

## RESULTS

The study’s subjects, both animals, survived the procedure. The mean duration of the procedure was 255 min, with a mean console time of 201 min. All anastomoses were performed without complications, with a mean anastomotic time of 90 min. The integrity of the anastomoses was evaluated for any bleeding or air leaks. The procedure was successfully completed via a closed-chest approach, utilizing 5 ports, and a subxiphoid incision with a closed wound guard to prevent gas leakage in all animals.

## CONCLUSIONS

The Da Vinci robotic platform has been evaluated in the experimental lab setting for a left single lung transplant with promising exposure and workflow. The visualization and ability to perform a precise dissection appears significantly improved over the existing approaches. This novel approach proved to be safe and feasible for lung transplantation in sheep and probably more beneficial for the patient than the CS incision in the setting of lung transplantation. Further studies in the clinical setting will be necessary to prove feasibility and safety in humans.

## Data Availability

All relevant data are within the manuscript and its Supporting Information files.
